# Generation of Insulin-Producing Cells from Human Bone Marrow-Derived Mesenchymal Stem Cells: Comparison of Three Differentiation Protocols

**DOI:** 10.1155/2014/832736

**Published:** 2014-04-10

**Authors:** Mahmoud M. Gabr, Mahmoud M. Zakaria, Ayman F. Refaie, Sherry M. Khater, Sylvia A. Ashamallah, Amani M. Ismail, Nagwa El-Badri, Mohamed A. Ghoneim

**Affiliations:** ^1^Department of Biotechnology, Urology and Nephrology Center, Mansoura 35516, Egypt; ^2^Department of Nephrology, Urology and Nephrology Center, Mansoura 35516, Egypt; ^3^Department of Pathology, Urology and Nephrology Center, Mansoura 35516, Egypt; ^4^Department of Immunology, Urology and Nephrology Center, Mansoura 35516, Egypt; ^5^Zewail University of Science and Technology, 6th of October City, Giza 12588, Egypt; ^6^Department of Urology, Urology and Nephrology Center, Mansoura 35516, Egypt

## Abstract

*Introduction*. Many protocols were utilized for directed differentiation of mesenchymal stem cells (MSCs) to form insulin-producing cells (IPCs). We compared the relative efficiency of three differentiation protocols. *Methods*. Human bone marrow-derived MSCs (HBM-MSCs) were obtained from three insulin-dependent type 2 diabetic patients. Differentiation into IPCs was carried out by three protocols: conophylline-based (one-step protocol), trichostatin-A-based (two-step protocol), and **β**-mercaptoethanol-based (three-step protocol). At the end of differentiation, cells were evaluated by immunolabeling for insulin production, expression of pancreatic endocrine genes, and release of insulin and c-peptide in response to increasing glucose concentrations. *Results*. By immunolabeling, the proportion of generated IPCs was modest (**≃**3%) in all the three protocols. All relevant pancreatic endocrine genes, insulin, glucagon, and somatostatin, were expressed. There was a stepwise increase in insulin and c-peptide release in response to glucose challenge, but the released amounts were low when compared with those of pancreatic islets. *Conclusion*. The yield of functional IPCs following directed differentiation of HBM-MSCs was modest and was comparable among the three tested protocols. Protocols for directed differentiation of MSCs need further optimization in order to be clinically meaningful. To this end, addition of an extracellular matrix and/or a suitable template should be attempted.

## 1. Introduction

Diabetes mellitus (DM) is a widespread devastating disease affecting millions of people worldwide. Maintaining good glycaemic control with exogenous insulin imposes a burden on patients. Transplantation of whole intact pancreas or isolated pancreatic islets is an alternative treatment for patients with type I DM. However, the shortages of cadaveric organs and the necessity to use immunosuppression are limiting factors [1].

Recent progress in the field of regenerative therapies has focused attention on generation of surrogate *β* cells from stem cells derived from embryonic, umbilical cord blood, Wharton's jelly, and a variety of adult tissues [2].

Human embryonic stem cells (h-ESCs) can be expanded and differentiated to all cell types including insulin-producing cells (IPCs) [3–5]. So far, the use of these cells is burdened by ethical considerations as well as by practical problems: the lack of available embryos, difficulties with the generation of immunocompatible cells, and the risk of uncontrolled malignant proliferation of residual undifferentiated cells. IPCs can also be obtained by directed molecular engineering of cells from umbilical cord [6]. These sources have the advantage of a large potential donor pool. Nevertheless, the risks of rejection and the need for immunosuppression remain as obstacles.

Bone marrow-derived mesenchymal stem cells (BM-MSCs) are multipotent and can differentiate into lineages of mesodermal, endodermal, and ectodermal cells [7]. This source provides an opportunity for generation of large numbers of autologous *β* cells without the major limitations of organ availability and/or allogenic rejection. In a previous study, we successfully treated diabetic Sprague Dawley rats with islet-like clusters derived from bone marrow of inbred donors [8]. More recently, we were able to generate IPCs from human bone marrow-derived mesenchymal stem cells (HBM-MSCs) using a three-step differentiation protocol [9]. However, the proportion of the generated IPCs was modest. Herein, we investigate the efficacy of two novel differentiation protocols and compare them to our previous protocol.

## 2. Material and Methods

### 2.1. Retrieval of Human Bone Marrow Cells

The required approvals for this study were obtained from the ethical committee of the University of Mansoura. Bone marrow aspirates (BMAs) were collected from iliac crests in heparin from 3 consenting donors. All the donors were type II, insulin-dependent diabetic patients (Table 1).

### 2.2. Isolation and Expansion of HBM-MSCs

The BMAs were diluted 1 : 1 with low glucose Dulbecco's Modified Eagle's Medium (DMEM, Sigma, St. Louis, Missouri, USA) layered atop a density gradient (Ficoll-Paque, 1.077 g/mL) (Pharmacia, Uppsala, Sweden) and centrifuged for 20 minutes at 600g. Cells were collected from the DMEM/Ficoll interface, washed twice in phosphate buffer saline (PBS), and resuspended in 10 mL of low glucose complete DMEM supplemented with 10% fetal bovine serum (Hyclone, Logan, UT, USA), 100 U/mL penicillin, and 100 U/mL streptomycin (Sigma). 1 mL of the BMA yielded ~1.5 × 10^6^ nucleated cells. The collected cells were cultured in complete DMEM at a density of 5 × 10^5^ cells/mL (10 mL in 25 cm^2^ tissue culture flasks) and incubated at 37°C in 5% CO_2_ incubator. Aliquots were preserved in liquid nitrogen for subsequent expansion and examination.

After 3 days, the nonadherent cells were discarded. The remaining adherent MSCs were cultured to 80% confluence before passaging with trypsin. Cells were resuspended with complete DMEM and replated at a ratio of 1 : 2 and cultured for a further 8 days, to reach 80% confluence. This step was repeated for a second passage. The cells then had the appearance of fibroblast-like cells.

### 2.3. Differentiation of HBM-MSCs into Endocrine Cells

At passage 3, MSCs were cultured at a density of 1 × 10^5^ cells/mL. Cells from each donor were induced to form IPCs using 3 different differentiation protocols.

#### 2.3.1. One-Step Protocol

This protocol was described previously by Hisanaga et al. [10]. The MSCs were cultured for 15 days in a high glucose (25 mM) DMEM containing 10% fetal bovine serum, 100 ng/mL conophylline (CnP) (kindly supplied by Prof. Kazuo Umezawa, Keio University, Yokohama, Japan), and 1 nM betacellulin-*δ*4 (BTC-*δ*4) (kindly supplied by Prof. Masaharu Seno, Okayama University, Japan).

#### 2.3.2. Two-Step Protocol

Differentiation was carried out according to the method previously reported by Tayaramma et al. [11]. Initially, cells were cultured for 3 days in serum-free DMEM supplemented with Trichostatin-A (TSA) at a concentration of 55 nM (Sigma-Aldrich Co., St. Louis, Missouri, USA). The cells were then cultured for additional 7 days in high glucose (25 mM) medium containing 1 : 1 ratio of DMEM : DMEM/F12 (Sigma). This mixture was supplemented with 10% fetal bovine serum and 10 nM glucagon-like peptide-1 (GLP-1, Sigma).

#### 2.3.3. Three-Step Protocol

We have previously utilized this protocol [9]. MSCs were cultured at a density of 1 × 10^5^ cells/mL in serum-free, high glucose DMEM (25 mM) containing 0.5 mM *β*-mercaptoethanol (Sigma) and incubated for 2 days. The medium was replaced with serum-free, high glucose DMEM containing 1% nonessential amino acids (Sigma), 20 ng/mL basic fibroblast growth factor (Sigma), 20 ng/mL epidermal growth factor (Sigma), 2% B27 supplement (Gibco BRL, Life Technologies, UK), and 2 mM L-glutamine (Sigma) and cultured for a further 8 days. Finally, the cells were cultured for additional 8 days in serum-free, high glucose DMEM containing 10 ng/mL betacellulin (Sigma), 10 ng/mL activin-A (Sigma), 2% B27 supplement, and 10 mM nicotinamide (Sigma).

### 2.4. Characterization of the Isolated HBM-MSCs

#### 2.4.1. Phenotyping

To ensure the exclusion of HBM-hematopoietic stem cells and purity of HBM-MSCs, cells at passage 3 weretrypsinized, centrifuged at 300g for 8 minutes, and resuspended in PBS at a concentration of 1 × 10^6^ cells/mL. 100 *μ*L aliquots were labeled (30 mins) with antibodies against CD14, CD45 (FITC) or CD73, CD34 phycoerythrin (PE) (Becton-Dickinson, USA), or CD105 PE or CD90 (FITC) (Becton-Dickinson, USA), washed with 1 mL of stain buffer (BD Pharmingen, USA), and resuspended in 500 *μ*L of stain buffer. The labeled cells were analyzed using an argon ion laser with a wave length of 488 nm (FACS Calibur, Becton-Dickinson, USA). A total of 10000 events were obtained and analyzed with the Cell Quest software program (Becton-Dickinson, USA). Control staining with appropriate isotype-matched monoclonal antibodies was included.

#### 2.4.2. Multilineage Differentiation Potential

HBM-MSCs were induced to differentiate into adipocytes, chondrocytes, and osteocytesusing a differentiation protocol as described previously [7]. Oil-Red-O was used to determine adipocytes phenotype, Alcian blue for chondrocytes, and alizarin-red for osteocytes.

### 2.5. Gene Expression by RT-qPCR

Total RNA was extracted from HBM-MSCs prior to and at the end of differentiation for all the samples according to the protocol employed using RNeasy Plus Mini Kit (Qiagen GmbH, Hilden, Germany). 3 *μ*g of total RNA was converted into cDNA using RT First Strand kit (Qiagen Sciences, Maryland, USA). Custom gene arrays were designed and supplied in 96-well plates (Qiagen Science, Maryland, USA). Gene expression was examined for endocrine hormones (insulin, glucagon, and somatostatin), transcription factors (PDX-1, Ngn 3, Pax 4, RFX6, and Neurod-1), endocrine precursor marker (nestin),  glucose transporter (Glut-2), and pancreatic enzyme (glucokinase). Human islets as well as GAPDH were also included to serve as positive and internal controls, respectively. Amplifications were performed in a 25 *μ*L reaction volume in each well that contains 12.5 *μ*L 2X SYBR Green Rox Master Mix (Qiagen Sciences, Maryland, USA), 1 *μ*L of cDNA template, and 11.5 *μ*L of nuclease-free water. The plate array was inserted in real time thermal cycler (ABI PRISM 7000, Applied Biosystem, California, USA) and programmed according to manufacturer instructions. For each sample, the procedure was carried out in duplicate. A mathematical model introduced by Pfaffl [12] was used for the relative quantification of target genes. In this study, gene expression was expressed relative to that of human islets.

### 2.6. Functional Evaluation of the Differentiated HBM-MSCs (DTZ Staining)

The ability of the differentiated HBM-MSCs to produce insulin was tested by staining with diphenylthiocarbazone (DTZ, zinc-chelating agent). Diphenylthiocarbazone 50 mg (Sigma) was dissolved in 5 mL dimethyl sulfoxide and stored at −20°C. For staining, 10 *μ*L of the stock solution was added to 1 mL of the culture medium. Cells were incubated at 37°C for 30 minutes in the prepared medium. Cells were then examined with a stereomicroscope after rinsing the plates 3 times with Hank's balanced salt solution (Sigma).

### 2.7. Immunolabeling

At the end of in vitro differentiation, cells were immunolabeled for their insulin content by flow cytometry and for their insulin, glucagon, and somatostatin by immunocytochemistry.

For flow cytometric analysis, cells at the end of in vitro differentiation were fixed in 4% formaldehyde for 10 minutes at 37°C, permeabilized by chilled 90% methanol for 30 minutes, and blocked by an incubation buffer (0.5 g bovine serum albumin in 100 mL PBS) for 10 minutes at RT. Cells were then incubated with the primary antibody for 30–60 minutes at RT (rabbit monoclonal anti-human insulin, Cell Signaling Technology). Cells were then washed by incubation buffer and after centrifugation were resuspended and incubated in fluorochrome-conjugated secondary antibody (polyclonal swine anti-rabbit IgG labeled with FITC 1 : 10, Dako Cytomation). Finally, cells were washed and resuspended in 0.5 mL PBS. The labeled cells were evaluated using an argon ion laser (15 mw) with a wave length of 488 nm (FACS Calibur, Becton-Dickinson, USA). A total of 10000 events were obtained and analyzed with the Cell Quest software program (Becton-Dickinson, USA). Human pancreatic islets served as a positive control.

For immunocytochemistry, these cell culture specimens on chamber slides (Nunc, Rochester, NY, USA) were fixed with 4% paraformaldehyde for 10 minutes at RT, permeabilized with 100% chilled methanol for 10 minutes, blocked with 5% normal goat serum for 60 minutes at RT, and incubated overnight with the primary antibodies at 4°C. These included mouse monoclonal anti-insulin, rabbit monoclonal anti-glucagon, rabbit polyclonal anti-c-peptide (cell Signaling Technology, Danvers, Mass., USA), and rabbit polyclonal anti-human somatostatin (Novus Biologicals Technology, Littleton, Colorado, USA). Subsequently, the cells were washed with PBS and incubated with secondary antibodies for 2 hours at RT (anti-mouse IgG (H + L) Alexa flour 488 conjugate and anti-rabbit IgG (H + L) Alexa flour 555 conjugate, Cell Signaling Technology). The nuclei were counterstained with DAPI (Invitrogen, UK). Negative controls were obtained by omitting treatment with the primary antibody. Confocal images were captured using Leica TCS SP8 microscope (Leica Microsystems, Mannheim, Germany).

### 2.8. Determination of In Vitro Insulin and c-Peptide Release in response to Increasing Glucose Concentrations

Four different samples of 1 × 10^6^ cells were collected from the same batch of each donor at the end of the differentiation period for measurement of released insulin and c-peptide hormones. Cells were initially incubated for 3 hours in glucose-free Krebs-Ringer bicarbonate buffer (KRB). This was followed by incubation for 1 hour in 3.0 mL of KRB containing 5.5, 12, or 25 mM glucose concentrations. The supernatant was collected at the end of each incubation period. The collected samples were frozen at −70°C until assayed using an Elisa Kit with a minimum detection limit of 1.76 *μ*IU/mL (DRG Diagnostic, Germany). Undifferentiated HBM-MSCs served as negative control.

### 2.9. Statistical Analysis

Nonparametric data were evaluated by Friedman's test. Post hoc testing was performed by Wilcoxon's Signed Ranking and the *P* values were corrected by Bonferroni adjustments. A *P* value of < 0.05 was considered significant.

## 3. Results

### 3.1. Morphological and Phenotypical Characterization of the Cultured HBM-MSCs

**Figure 1:**
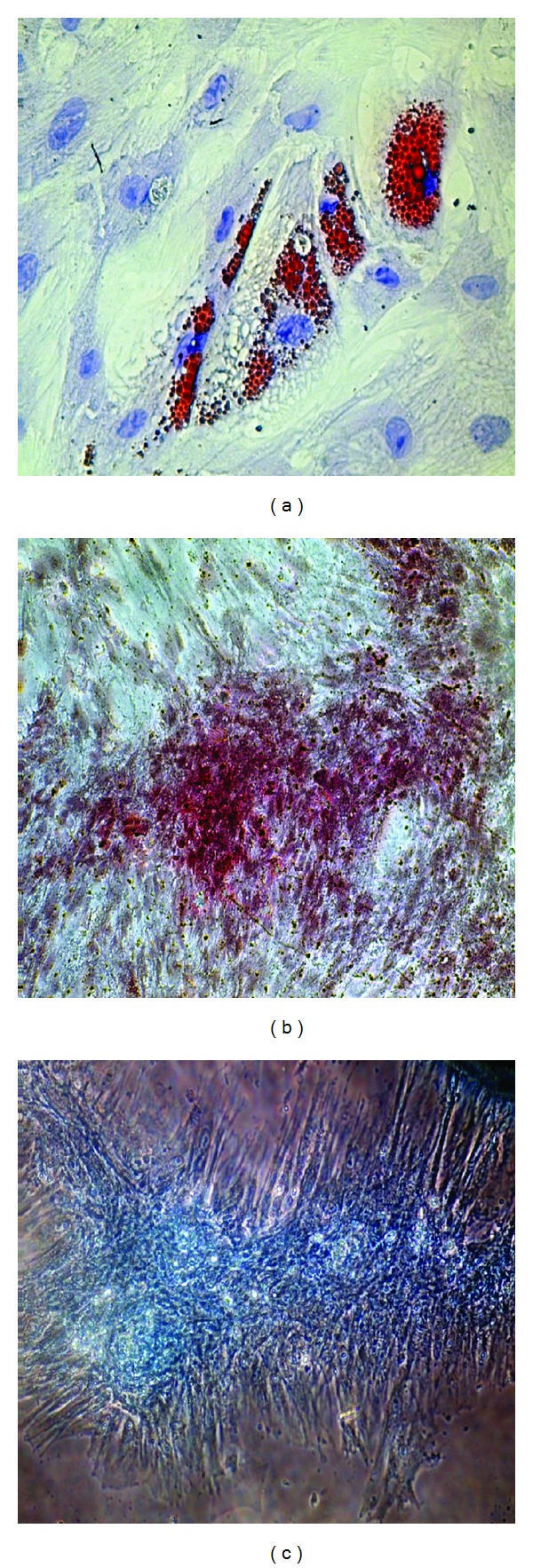
Multilineage differentiation of HBM-MSCs. (a) Adipogenesis was detected using Oil-Red-O staining. (b) Osteogenesis was detected using alizarin-red staining. (c) Chondrogenesis was detected using Alcian blue.

At the end of the expansion phase, the cultured human bone marrow cells become homogenous, spindle shaped, and fibroblast-like arranged in monolayers. There were no apparent differences in the rate of duplication of cells derived from the three donors. Flow cytometric analysis showed that these cells expressed high levels of CD73, CD90, and CD105 but negligible levels of CD14, CD34, and CD45, which are surface markers for hematopoietic stem cells (Table 2). Moreover, their multilineage differentiation potential was confirmed. The cells could be differentiated to form adipocytes, chondrocytes, and osteocytes when the appropriate growth factors were added. These data indicate that the majority of the human bone marrow-derived cells were MSCs (Figure 1).

### 3.2. Functional Evaluation of the Differentiated HBM-MSCs

**Figure 2:**
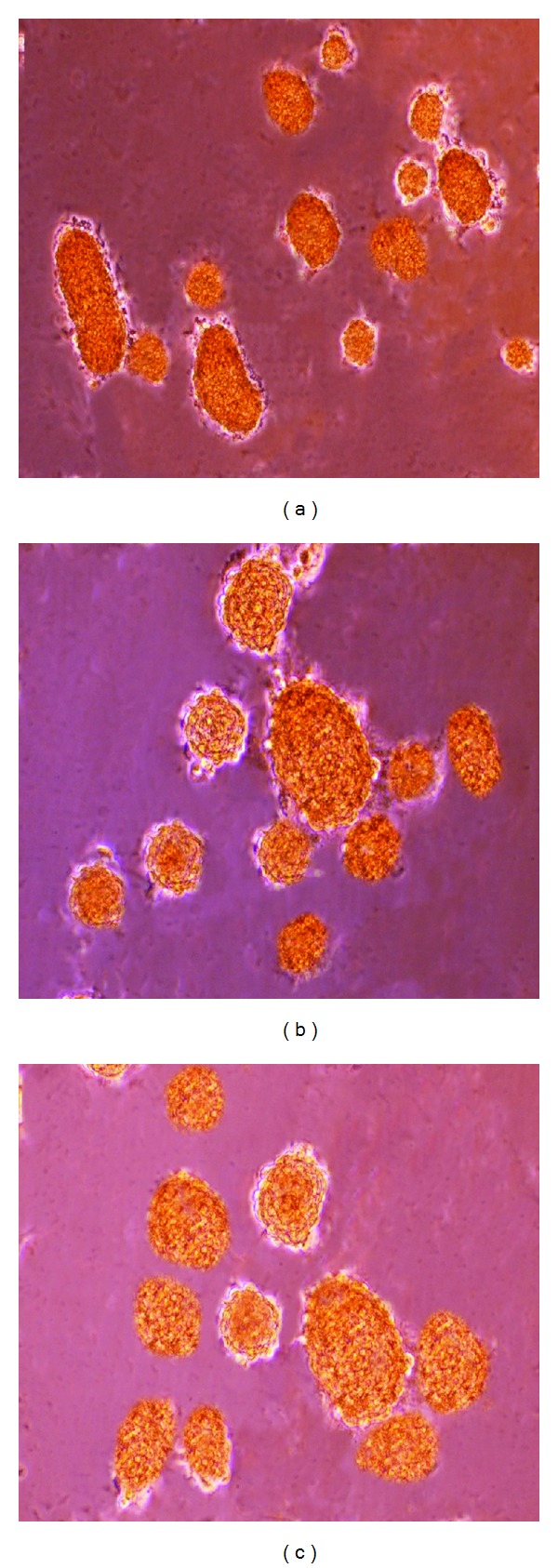
DTZ staining of HBM-MSCs at the end of in vitro differentiation. (a) One-step protocol. (b) Two-step protocol. (c) Three-step protocol.

The differentiated HBM-MSCs obtained from the three differentiation protocols were distinctly stained crimson red by DTZ (Figure 2).

### 3.3. Immunolabeling

At the end of differentiation, flow cytometric analysis indicated that the proportion of insulin-positive cells generated by the three differentiation protocols was essentially similar and ranged between 0.7% and 5%. However, within the same protocol, there was an interdonor variability though not significant (Table 3). Glucagon staining was positive in some of the studied samples. However, positive staining for somatostatin was not seen.

**Figure 3:**
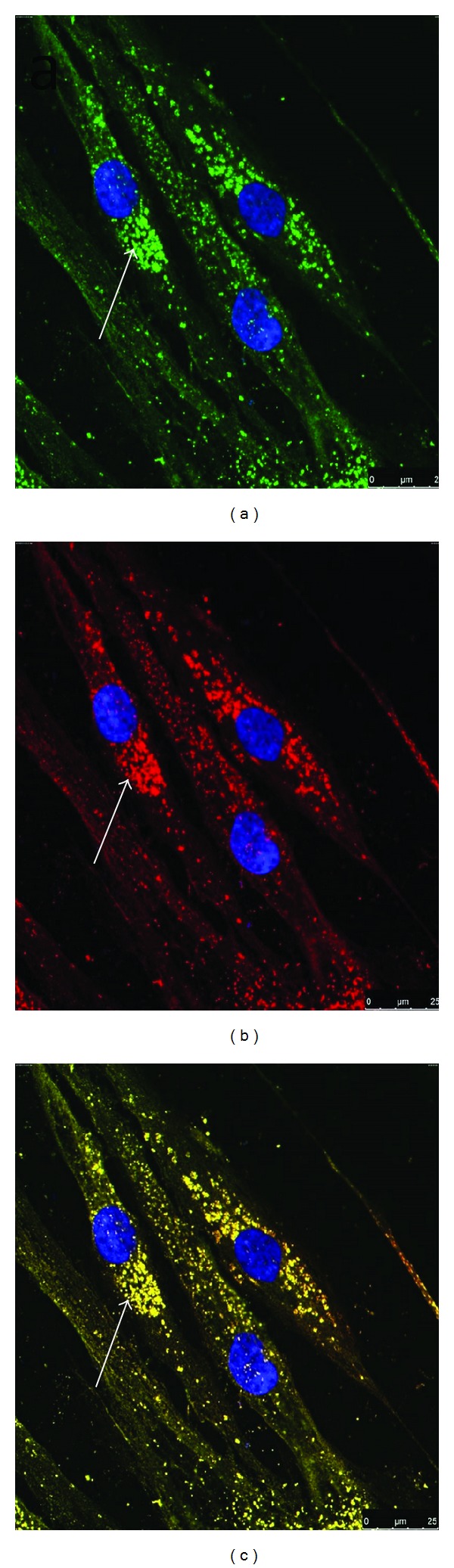
Immunofluorescent staining of differentiated HBM-MSCs using the TSA-based protocol. (a) Positive staining for insulin (green) with counterstaining for DAPI. (b) Positive staining for c-peptide (red) with counterstaining for DAPI. (c) Electronic merge of insulin and c-peptide. Coexpression of insulin and c-peptide (yellow) by the same cells could be seen.

With immunocytochemistry, the presence of insulin granules within the cytoplasm of the insulin-positive cells was seen. In addition, immunostaining for c-peptide was also positive. Coexpression of insulin and c-peptide within the same cells was observed following electronic merging (Figure 3).

### 3.4. Gene Expression by RT-qPCR

**Figure 4:**
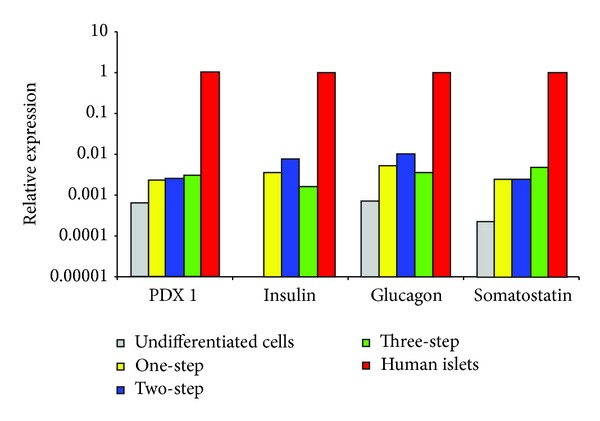
Relative gene expression at the end of in vitro differentiation comparing the three differentiation protocols (donor # 1). In the undifferentiated MSCs, insulin gene was not expressed, while gene expression for PDX-1, glucagon, and somatostatin was minimal. At the end of in vitro differentiation by all of the three protocols, the relevant endocrine genes, insulin, glucagon, and somatostatin, were expressed. Relative expression of insulin and glucagon was more prominent with the two-step protocol (TSA-based). Following this protocol, PDX-1 gene expression was increased by 4-fold, insulin gene expression by 2500-fold, glucagon gene expression by 14-fold, and somatostatin gene expression by 10-fold, when compared to the undifferentiated MSCs.

In the undifferentiated MSCs, insulin gene was not expressed, while gene expression for PDX-1, glucagon, and somatostatin was minimal. At the end of in vitro differentiation by all of the three protocols, the relevant endocrine genes, insulin, glucagon, and somatostatin, were expressed. Relative expression of insulin and glucagon was more prominent with the two-step protocol (TSA-based). Following this protocol, insulin gene was clearly expressed, PDX-1 gene expression was increased by 4-fold, glucagon gene expression was increased by 14-fold, and somatostatin gene expression was increased by 10-fold, when compared to the undifferentiated MSCs (Figure 4). The numerical values for relative gene expression values for the studied genes of cells from all donors and by the three protocols are provided (see supplementary Tables 1, 2, and 3, in Supplementary Material available online at http://dx.doi.org/10.1155/2014/832736).

### 3.5. In Vitro Human Insulin and c-Peptide Release in response to a Glucose Challenge

**Figure 5:**
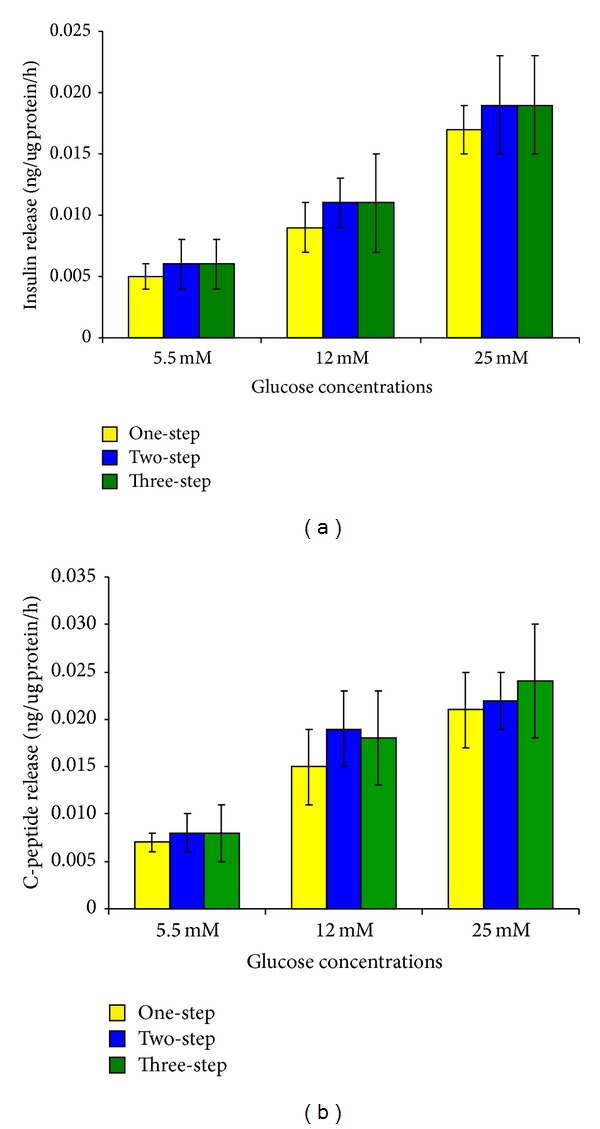
In vitro human insulin and c-peptide release in response to glucose challenge. The differentiated cells obtained by the three studied protocols releasedincreasing amounts of insulin (a) and c-peptide (b) in response to increasing glucose concentrations (*P* < 0.05). The amounts of insulin and c-peptide released at different concentrations of glucose were comparable among the three experimental groups.

The differentiated cells obtained by the three studied protocols released increasing amounts of insulin and c-peptide in response to increasing glucose concentrations (*P* < 0.05). The amounts of insulin and c-peptide released at different concentrations of glucose were comparable among the three experimental groups (Figure 5). There was no insulin nor c-peptide release by the undifferentiated HMB-MSCs. The numerical values for released amounts of insulin and c-peptide by the three protocols are provided (Tables 4 and 5, supplementary data).

## 4. Discussion

MSCs are self-renewing cells with a multilineage differentiation potential [7]. Their ease of isolation and expansion has rendered them a potentially important source of stem cells for the use in tissue engineering and regenerative medicine including a therapeutic potential for DM [13]. MSCs could be obtained from different adult human tissues such as bone marrow and adipose tissue. Recently, it was reported that bone marrow-derived MSCs are superior to those obtained from subcutaneous adipose tissue in terms of differentiation into IPCs [14].

Many protocols have been studied to induce differentiation of stem cells obtained from different sources to form IPCs [15, 16]. We had previously shown that MSCs derived from adult human bone marrow can be isolated, expanded, and differentiated using a three-step protocol [9]. However, the proportion of generated IPCs was modest. In an attempt to increase the proportion and optimize insulin production, the current study was designed to compare the efficiency of three different protocols for their differentiation potentials into IPCs.

In this study, HBM-MSCs were isolated on the basis of their ability to adhere to plastic. At the end of expansion, these cells assumed a spindle-shaped morphology and were negative for hematopoietic cell markers. Furthermore, their differentiation into adipocytes, chondrocytes, and osteocytes confirmed their multilineage potentials. The initial step in directed differentiation to form IPCs is to induce expression of PDX-1. PDX-1 is one of the well-studied transcription factors that are critical to both *β* cell development and function [17]. PDX-1 controls the maturation of pancreatic islet cells by regulating insulin 1 and other downstream genes. It was shown that the expression of PDX-1 is switched on first followed by other IPCs-related genes [18]. In the current study, we have utilized Cnp in the one-step protocol, TSA in the two-step protocol, and *β*-mercaptoethanol in the three-step protocol to induce expression of PDX-1.

CnP is a novel differentiation inducer for pancreatic *β* cells [19] through activation of P38 nitrogen-activated protein kinase [20], which plays a critical role in inducing neurogenin-3 gene [21]. BTC plays an important role in regulating growth and/or differentiation of pancreatic endocrine precursor cells. Acting in coordination on the different steps of differentiation, CnP and BTC-*δ*4 augment differentiation of *β* cells in vitro [22].

The actual chromatin structure and its remodeling are an important mode of the epigenetic control of gene expression [23]. TSA is a natural product isolated from the metabolites of strains of* Streptomyces hygroscopicus* with antifungal and antibiotic activities [24]. Evidences were provided that TSA has the potential of chromatin remodeling and can allow differentiation of bone marrow cells into IPCs under appropriated culture conditions in the presence of high glucose concentrations and GLP-1 [11]. Glucose is well known as a growth factor for *β* cells [17]. It promotes *β* cells replication in vitro as well as in vivo at concentrations of 20–30 mmol/L [25]. GLP-1 is an incretin hormone capable of converting intestinal epithelial cells into functional IPCs [26]. It should be noted that the one-step protocol (CnP-based) as well as the two-step protocol (TSA-based) had previously used bone marrow-derived cells from mice. In this study, we report, for the first time, the utilization of such protocols for the differentiation of HBM-MSCs to form IPCs.

In the third protocol, *β*-mercaptoethanol was used to induce expression of transcription factor, PDX-1 [27]. Subsequently, fibroblast and the epidermal growth factors were used, since these were shown in an experimental model to stimulate proinsulin biosynthesis and 3H-thymidine incorporation [28]. Activin-A regulates the exogenesis of *β* cells in vivo, while BTC plays an important role in regulating growth and/or differentiation of pancreatic endocrine precursor cells [29]. Nicotinamide is an effective inducer used to preserve islet viability through poly-ADP-ribose polymerase (PARP) [30].

The insulin gene was not detected in the undifferentiated MSCs. At the end of in vitro differentiation by the three studied protocols, pancreatic endocrine genes were readily expressed. This was particularly evident with the two-step protocol (TSA-based). However, this expression was far lower than that of the human islets which were used as a positive control. This may explain the modest proportion of the generated IPCs and the low levels of insulin and c-peptide release when compared to those of the isolated pancreatic islets [31, 32]. These findings were also reported by several investigators [33, 34]. Karnieli et al. suggested that the absence of expression of Neurod-1 at the end of the in vitro differentiation is likely a major cause for the incomplete *β* cell phenotype and consequently the inefficient glucose-stimulated insulin secretion [35]. However, in our experiments and with the three utilized protocols, Neurod-1 was expressed. We postulate that directed differentiation would induce an increased gene transcription but not reaching the threshold to generate synthesis of sufficient amounts of insulin within the cytoplasm that can be detected by immunolabeling.

The poor insulin release in response to glucose challenge was also reported by several investigators [35, 36]. Comparisons between the reported data are difficult since different units of measurements were used for reference. Kim et al. [37] compared insulin release by differentiated human MSCs derived from various sources. At a concentration of 25 mM of glucose, insulin release ranged between 4 and 16 ng/mg protein (0.004–0.016 ng/ug). Wei et al. [38] reported that insulin release by differentiated human embryonic cells at a glucose concentration of 16.7 mm ranged between 200 and 900 *μ*IU/mg protein (0.008–0.04 ng/ug). Ilie et al. reported that insulin release by human *β* cells at 15 mM glucose concentration was approximately 0.27 ng/1 × 10^6^ cells/hour [39]. Our data indicate that, for a similar number of cells, insulin release at an equivalent glucose concentration was 0.009 ng/ug/hour. Accordingly, this amount corresponds to roughly 3% of that released by human islets. This ratio is supported by the findings of Karnieli et al. [35]. They have estimated the insulin content of insulin-producing cells derived from HBM-MSCs as 1% of that of normal human islets. In spite of these marginal data, we have previously reported that transplantation of 1 × 10^6^ of HBM-MSCs differentiated by the three-step protocol was able to induce normoglycemia in diabetic nude mice [9].

## 5. Conclusion

So far, in our experience and that of others, directed differentiation of HBM-MSCs to form functional IPCs needs further optimization in order to be clinically meaningful. In our future experiments, it is planned to use the TSA-based differentiation protocol in view of its simplicity and the short duration needed for differentiation. To this end, addition of an extracellular matrix and/or a suitable template should be attempted. Despite the modest percent of insulin-positive cells at the end of in vitro differentiation, they were able to induce normoglycemia after their transplantation in diabetic nude mice. This paradoxical finding would certainly require an explanation and is a subject matter of a current study.

## Supplementary Material

Tables 1, 2, and 3 provide the numerical values of relative gene expression of HBM-MSCs obtained from the 3 donors: at the end of expansion as well as at the end of differentiation. The tables also provide a comparison between the 3 studied differentiation protocols; values of human islets are used for reference.
Values of in vitro released human insulin (table 4) and c-peptide (table 5) as a function of a glucose challenge by the differentiated HBM-MSCs obtained from the three donors with a comparison between the 3 studied differentiation protocols.

## Figures and Tables

**Table 1:** Characteristics of the bone marrow diabetic volunteers.

Donor	Age (years)	Sex	Duration of DM (years)	Postprandial c-peptide (ng/mL)	HbA1_c_ (%)	Insulin dose (IU/day)
1	57	Male	10	2.7	11.9	50
2	53	Male	4	0.7	7.2	45
3	35	Female	5	1.6	11.4	90

**Table 2:** Flow cytometric quantitation of surface markers of the undifferentiated HBM-MSCs.

Donor	CD14 (%)	CD34 (%)	CD45 (%)	CD73 (%)	CD90 (%)	CD105 (%)
1	3.9	1.1	0.9	97.9	95.6	91.6
	2.0	0.1	0.6	95.1	87.4	92.3
	2.1	0.5	0.4	96.2	95.3	93.5

2	0.7	2.7	0.4	98.4	98.5	98.3
	0.9	1.4	0.8	99.4	99.5	98.9
	0.7	1.3	2.7	87.1	95.2	95

3	3.2	0.1	0.2	96.7	88.3	94.5
	0.7	0.9	0.1	97.8	95.5	98.6
	1.5	1.6	0.4	96.7	98.4	91.6

Mean ± S.D.	1.7 ± 1.1	1.1 ± 0.8	0.7 ± 0.6	97.3 ± 1.3	94.9 ± 4.3	94.9 ± 2.9

**Table 3:** Insulin positive cells generated by the three protocols (%).

Protocol	One-step	Two-step	Three-step
Donor 1	2.6%	1.8%	0.4%
	2.6%	1.4%	0.43%
	4.0%	1.9%	3.2%

Donor 2	2%	3.4%	0.98%
	1.8%	5.0%	2.1%
	2.1%	1.0%	3.5%

Donor 3	1%	1.47%	0.96%
	1.4%	2.80%	3.50%
	0.7%	3.60%	3.9%

Mean ± S.D.	2.02 ± 0.98	2.49 ± 1.31	2.11 ± 1.44

*P* > 0.05.
