# Supplementary material for: Generation of Insulin-Producing Cells from Human Bone Marrow-Derived Mesenchymal Stem Cells: Comparison of Three Differentiation Protocols
Source: Biomed Res Int. 2014 Apr 10;2014:832736. doi: 10.1155/2014/832736 (PMC4000976; doi:10.1155/2014/832736)
Supplement: Supplementary file 1 — Tables 1, 2, and 3 provide the numerical values of relative gene expression of HBM-MSCs obtained from the 3 donors: at the end of expansion as well as at the end of differentiation. The tables also provide a comparison between the 3 studied differentiation protocols; values of human islets are used for reference. Values of in vitro released human insulin (table 4) and c-peptide (table 5) as a function of a glucose challenge by the differentiated HBM-MSCs obtained from the three donors with a comparison between the 3 studied differentiation protocols. [file 832736.f1.pdf]

Table 1: Values for relative gene expression values for the studied genes of HBM-MSCs by the three studied protocols (donor# 1)

	Undifferentiated MSCs		One-Step		Two-Step		Three-Step		Human Islets
**INS**	Undetected	Undetected	0.00352	0.00354	0.008609	0.006801	0.001724	0.001642	1
**GCG**	0.00072	0.00066	0.00427	0.00630	0.010525	0.009099	0.003988	0.003217	1
**PDX1**	0.00061	0.00065	0.00290	0.00175	0.002743	0.002355	0.003262	0.002705	1
**SST**	0.00024	0.00021	0.00251	0.00229	0.002388	0.002323	0.005263	0.003906	1
**NGN3**	0.00045	0.00000	0.00729	0.00787	0.00194	0.001289	0.005563	0.010453	1
**PAX4**	0.00016	0.00015	0.00148	0.00430	0.00072	0.000569	0.001874	0.001381	1
**GCK**	0.00370	0.00357	0.00694	0.01584	0.005119	0.004979	0.001228	0.001099	1
**RFX6**	0.00246	0.00278	0.01305	0.01871	0.007599	0.010097	0.006434	0.007599	1
**GLUT2**	0.00058	0.00047	0.00306	0.00962	0.003721	0.003799	0.002388	0.002137	1
**Neurod1**	0.00035	0.00039	0.03280	0.04904	0.11908	0.119908	0.035403	0.021344	1
**NES**	0.00319	0.00261	0.02149	0.02936	0.018453	0.017948	0.00734	0.006661	1
**MafA**	0.00239	0.00249	0.00989	0.01128	0.003377	0.004876	0.007759	0.003496	1
**MafB**	0.00704	0.00609	0.01031	0.01418	0.007041	0.007041	0.200267	0.205898	1
**OCT4**	0.00043	0.00042	0.00526	0.00996	0.000899	0.000918	0.002507	0.002472	1

Table 2: Values for relative gene expression values for the studied genes of HBM-MSCs by the three studied protocols (donor # 2)

	Undifferentiated MSCs		One-Step		Two-Step		Three-Step		Human Islets
**INS**	Undetected	Undetected	0.005119	0.006848	0.00271	0.00328	0.006708	0.005839	1
**GCG**	0.000662	0.00075	0.001575	0.001677	0.00267	0.00267	0.008609	0.004334	1
**PDX1**	0.000745	0.000905	0.001186	0.000937	0.00229	0.00160	0.004158	0.003773	1
**SST**	0.002291	0.003354	0.002455	0.004187	0.00132	0.00436	0.007546	0.006661	1
**NGN3**	0.004809	0.005486	0.002275	0.003065	0.00189	0.00254	0.013602	0.011359	1
**PAX4**	0.000549	0.000772	0.000386	0.000386	0.00040	0.00072	0.002542	0.002631	1
**GCK**	0.000103	9.12E-05	0.000269	0.00016	0.00065	0.00064	0.001511	0.00141	1
**RFX6**	0.005524	0.006215	0.001069	0.002152	0.00218	0.00286	0.004072	0.004187	1
**GLUT2**	0.000394	0.000557	0.001298	0.0014	0.00302	0.00413	0.003961	0.003472	1

**Neurod1**	0.007759	0.00613	0.008032	0.008912	0.01278	0.04972	0.021197	0.020193	1
**NES**	0.004876	0.002182	0.006302	0.005263	0.01468	0.02336	0.009486	0.008609	1
**MafA**	0.000459	0.000681	0.00113	0.001586	0.00313	0.00385	0.005226	0.010097	1
**MafB**	0.001631	0.001501	0.007867	0.007442	0.00249	0.00340	0.009037	0.007867	1
**OCT4**	0.000316	0.000229	0.000343	0.00044	0.00071	0.00112	0.002388	0.001913	1

Table 3: Values for relative gene expression values for the studied genes of HBM-MSCs by the three studied protocols (donor # 3)

	**Undifferentiated MSCs**		**One-Step**		**Two-Step**		**Three-Step**		**Human Islets**
**INS**	Undetected	Undetected	0.00530	0.00498	0.00568	0.00523	0.002093	0.002899	1
**GCG**	0.00003	0.00003	0.00662	0.01252	0.00734	0.00916	0.004581	0.005048	1
**PDX1**	0.00001	0.00001	0.00523	0.00626	0.00744	0.01192	0.002613	0.003044	1
**SST**	0.01184	0.01305	0.00820	0.00916	0.00803	0.00891	0.006661	0.006992	1
**NGN3**	0.01782	0.01951	0.00600	0.00798	0.01698	0.01618	0.007239	0.008851	1
**PAX4**	0.00803	0.00949	0.00170	0.00180	0.00227	0.00439	0.00198	0.002613	1
**GCK**	0.00015	0.00022	0.00622	0.00699	0.00155	0.00407	0.001298	0.0017	1
**RFX6**	0.04389	0.05329	0.026461	0.084788	0.01024	0.03955	0.003906	0.004776	1
**GLUT2**	0.00362	0.00362	0.00290	0.00302	0.00465	0.00494	0.004809	0.003879	1
**Neurod1**	0.05403	0.06381	0.04870	0.02664	0.03443	0.02486	0.071298	0.019104	1
**NES**	0.00220	0.00204	0.00512	0.00719	0.00744	0.00549	0.275476	0.325335	1
**MafA**	0.00126	0.00129	0.01017	0.00837	0.00910	0.00765	0.005263	0.006346	1
**MafB**	0.00635	0.00729	0.00110	0.00091	0.00244	0.00212	0.156041	0.154963	1
**OCT4**	0.00046	0.00043	0.00160	0.00319	0.00357	0.00328	0.002137	0.002323	1

Table (4): Values for In vitro human insulin release (ng/µg protein/hour) among cells from all donors in the three protocols

Glucose conc. / Protocol	5.5 mM	12 mM	25 mM	P-Value
**One Step:**				
**Donor 1**	0.005	0.009	0.018	
	0.003	0.007	0.015	
	0.003	0.008	0.017	
	0.004	0.009	0.019	
	---------------------	----------------------	---------------------	
	0.004	0.01	0.016	
**Donor 2**	0.007	0.012	0.018	
	0.006	0.011	0.019	
	0.007	0.013	0.02	
	---------------------	----------------------	---------------------	
	0.004	0.009	0.015	
**Donor 3**	0.003	0.008	0.012	
	0.006	0.01	0.02	
	0.004	0.008	0.017	
**Mean ± S.D**	**0.005 ± 0.001**	**0.009 ± 0.002**	**0.017 ± 0.002**	**< 0.05**

**Two Step:**				
**Donor 1**	0.007	0.01	0.02	
	0.005	0.008	0.019	
	0.008	0.012	0.024	
	0.005	0.009	0.02	
	---------------------	-----------------------	---------------------	
**Donor 2**	0.007	0.013	0.024	
	0.008	0.015	0.026	
	0.005	0.012	0.019	
	0.007	0.014	0.021	
	---------------------	-----------------------	---------------------	
**Donor 3**	0.003	0.009	0.01	
	0.005	0.01	0.016	
	0.007	0.012	0.02	
	0.004	0.01	0.02	
**Mean ± S.D**	**0.006 ± 0.002**	**0.011 ± 0.002**	**0.019 ± 0.004**	**< 0.05**

**Three Step:**				
**Donor 1**	0.003	0.005	0.014	
	0.005	0.008	0.017	
	0.002	0.004	0.012	
	0.003	0.006	0.013	
	---------------------	-----------------------	---------------------	
**Donor 2**	0.006	0.013	0.019	
	0.009	0.015	0.022	
	0.005	0.011	0.018	
	0.007	0.013	0.02	
	---------------------	-----------------------	---------------------	
**Donor 3**	0.008	0.014	0.023	
	0.006	0.012	0.019	
	0.009	0.016	0.025	
	0.008	0.015	0.023	
**Mean ± S.D**	**0.006 ± 0.002**	**0.011 ± 0.004**	**0.019 ± 0.004**	**< 0.05**
**P-Value**	**0.23**	**0.02**	**0.05**	

Table (5): Values for In vitro human c-peptide release (ng/µg protein/hour) among cells from all donors in the three protocols

Glucose conc. / Protocol	5.5 mM	12 mM	25 mM	P-Value

**One Step:**				
	0.007	0.015	0.02	
**Donor 1**	0.006	0.012	0.018	
	0.005	0.013	0.017	
	0.006	0.016	0.022	
	----------------------	--------------------------	-----------------------	
	0.006	0.011	0.018	
**Donor 2**	0.009	0.02	0.027	
	0.008	0.018	0.023	
	0.009	0.02	0.028	
	----------------------	--------------------------	-----------------------	
	0.006	0.012	0.02	
**Donor 3**	0.005	0.01	0.018	
	0.008	0.017	0.025	
	0.005	0.011	0.02	
**Mean ± S.D**	**0.007 ± 0.001**	**0.015 ± 0.004**	**0.021 ± 0.004**	**< 0.05**

**Two Step:**				
**Donor 1**	0.009<br>0.007<br>0.01<br>0.006	0.018<br>0.015<br>0.02<br>0.013	0.023<br>0.02<br>0.028<br>0.019	
	----------------------	-------------------------	----------------------	
**Donor 2**	0.009<br>0.01<br>0.007<br>0.008	0.018<br>0.02<br>0.015<br>0.019	0.023<br>0.027<br>0.021<br>0.024	
	----------------------	-------------------------	----------------------	
**Donor 3**	0.005<br>0.007<br>0.008<br>0.006	0.01<br>0.017<br>0.019<br>0.01	0.018<br>0.022<br>0.023<br>0.019	
**Mean ± S.D**	**0.008 ± 0.002**	**0.019± 0.004**	**0.022 ± 0.003**	**< 0.05**

Three Step:				
**Donor 1**	0.005	0.012	0.018	
	0.007	0.014	0.02	
	0.004	0.011	0.016	
	0.005	0.013	0.018	
	----------------------	--------------------------	-----------------------	
**Donor 2**	0.009	0.02	0.026	
	0.01	0.022	0.03	
	0.007	0.016	0.021	
	0.009	0.02	0.029	
	----------------------	------------------------	---------------------	
**Donor 3**	0.01	0.023	0.03	
	0.008	0.017	0.024	
	0.012	0.024	0.032	
	0.011	0.023	0.03	
**Mean ± S.D**	**0.008 ± 0.003**	**0.018 ± 0.005**	**0.024 ± 0.006**	**< 0.05**
**P-Value**	**0.39**	**0.32**	**0.33**	
